# Supplementary material for: Expanding Genetic Code to Generate Human Brain Organoids with Both Vasculature and Microglia‐Like Cells
Source: Adv Sci (Weinh). 2026 Sep 29:e77668. Online ahead of print. doi: 10.1002/advs.77668 (PMC13621534; doi:10.1002/advs.77668)
Supplement: Supplementary file 1 [file ADVS-9999-e77668-s001.docx]

**Supplemental Information**

**Expanding genetic code to generate human brain organoids with both vasculature and microglia-like cells**

Haishuang Lin^1,2#*^, Yaqing Wang^1,2#^, Hu Du^1,2^, Yuxin Qin^1,2^, Haoyu Zhang^1,2^, Peng Wang^1,2^, Lin Wei^3^, Jianhua Qin^1,2,4,5*^

^1^School of Biomedical Engineering, Division of Life Sciences and Medicine, University of Science and Technology of China, Hefei, China.

^2^Suzhou Institute for Advanced Research, University of Science and Technology of China, Suzhou, China.

^3^School of Life Sciences, Anhui Medical University, Hefei, China.

^4^Division of Biotechnology, Dalian Institute of Chemical Physics, Chinese Academy of Sciences, Dalian, China.

^5^University of Chinese Academy of Sciences, Beijing, China.

\# These authors contributed equally to this work.

Corresponding author: hlin9@ustc.edu.cn; [jhqin@dicp.ac.cn](mailto:jhqin@dicp.ac.cn)

**Supplementary Figures**

**Supplementary Figure 1. Characterization of hPSCs harboring bio-orthogonal genetic translation machinery (GTM).**

(**a**) Bright-field images showing stable H9^GTM^ lines during screening and passaging. Scale bar, 50 μm.

(**b**) Immunostaining of pluripotency markers OCT4, NANOG, ALP, and SSEA4 in H9^GTM^ cells. Scale bars, 50 μm.

(**c, d**) Quantification of total cell number (**c**) and percentage of viable cells (**d**) in H9-hESCs and H9^GTM^ cells. Data are shown as mean ± SD (n = 3 from three independent batches).

(**e**) Embryoid body differentiation assay showing H9^GTM^-derived NESTIN+ ectodermal, Brachyury+ mesodermal, and FOXA2+ endodermal cells. Scale bar, 50 μm.

**Supplementary Figure 2. Regulation of endothelial differentiation in H9^GTM^ cells.**

(**a**) Schematic of stable H9^GTM^ cell line generation and subsequent induction of endothelial differentiation by NAEK treatment for 2 days.

(**b**) Bright-field images showing morphological changes in H9^GTM^ cells following NAEK treatment. Scale bar, 50 μm.

(**c**) Quantitative RT-PCR analysis of *ETV2* and the endothelial marker gene *VE-Cadherin*. Data are shown as mean ± SD (n = 3 from three independent batches).

**Supplementary Figure 3. NAEK treatment induces morphological changes in vhCOs by day 6.** (a) Comparative bright-field images showing morphological differences between hCOs and vhCOs at day 6 of NAEK treatment. Scale bar, 150 μm. (b) LDH assays showing no significant cell death between hCOs and vhCOs at day 6 of NAEK treatment on day 8 and 10.

**Supplementary Figure 4. Characterization of vascular networks and marker expression in vhCOs.**

(**a**) Immunostaining of CD31 and COL4 in different organoids, revealing a rich vascular network. Scale bar, 25 μm.

(**b**) Mean fluorescence intensity of CD31 in different batches of vhCOs.

(**c**) Whole-mount immunostaining of CD31 and SOX2 in a day-30 vhCO. Scale bar, 100 μm.

(**d**) Triple immunostaining (CD31, PDGFRβ, GFAP) showed astrocytes in close association with the pericyte–endothelial vascular network. Scale bar, 100 μm.

(**e**) Quantification of pericyte coverage, CD31/ZO-1 colocalization and astrocyte coverage.

(**f**) Quantitative RT-PCR analysis of endothelial (*CD31, CD144*), pericyte (*PDGFRβ*), microglial (*IBA1*), astrocyte (*S100β, GFAP*), pluripotency (*OCT3/4, NANOG*), GTM system (*ETV2, MmPylRS*), and vasculogenesis-related genes (*MME, GLUT1*) at indicated time points (**Related to Fig. 2q**). Data are shown as mean ± SD (n = 3 from three independent batches).

(**f**) Negative control staining. A day-30 brain organoid cultured without NAEK was immunostained for CD31, SOX2, IBA1, S100β, and PDGFRβ. Scale bar, 100 μm.

(**g**) A non-GTM control treated with NAEK showed no CD31expression.

(**h**) Immunostaining for CD31 at day 90 and day 180 in the absence of NAEK.

**Supplementary Figure 5.** (**a**) Astrocyte-like cells exhibiting AQP4-labeled end-feet structures in day-90 vhCOs. Scale bar, 50 μm.

(**b**) Endothelial cells and pericytes organized into a typical cytoarchitectural pattern in day-90 vhCOs. Scale bar, 20 μm.

**Supplementary Figure 6. Characterization of microglia in vhCOs.**

(**a-c**) IBA1+ cells in vhCOs from three independent batches. Scale bar, 50 μm.

(**d**) Quantification of IBA1+ cells normalized to DAPI+ nuclei across the three batches shown in (**a-c**). Data are shown as mean ± SD (n = 3 from three independent batches).

(**e**) Immunostaining for CD31 and CD68 revealing activated microglia in close proximity to the vascular network. Scale bar, 50 μm.

(**f**) Expression of microglia-specific markers P2RY12, TREM2, and TMEM119 in day-90 vhCOs. Scale bar, 50 μm.

(**g, h**) Functional response of microglia to LPS stimulation. (**g**) Schematic of LPS treatment: day-90 vhCOs were treated with or without 100 ng/mL LPS for 24 hours. (**h**) ELISA analysis of culture supernatant showing significantly increased TNF-α and IL-6 secretion in LPS-stimulated vhCOs. Data are shown as mean ± SD (n = 3 from three independent batches).

**Supplementary Figure 7. Synaptic density in hCOs and vhCOs at day 90.**

(**a**) Immunostaining for TUJ1 (neuronal marker) and Synapsin (synaptic marker) in day-90 hCOs and vhCOs. Scale bar, 100 μm.

(**b**) Quantification of Synapsin mean fluorescence intensity in day-90 hCOs and vhCOs. Data are shown as mean ± SD (n = 3 from three independent batches).

**Supplementary Figure 8. Reduced apoptosis in vhCOs compared to hCOs.**

(**a, b**) TUNEL staining (**a**) and quantification (**b**) showing decreased apoptosis in vhCOs relative to hCOs. Scale bar, 100 μm. Data are shown as mean ± SD (n = 3 from three independent batches).

(**c-e**) Co-immunostaining for TUNEL and CD31 (**c, d**) and quantification (**e**) confirming reduced apoptosis in vhCOs compared to hCOs. Scale bars, 200 μm. Data are shown as mean ± SD (n = 3 from three independent batches).

**Supplementary Figure 9. Characterization of hCO differentiation from H9 cells.**

(**a**) Schematic diagram illustrating the developmental stages of human cerebral organoid (hCO) differentiation.

(**b-d**) Immunostaining confirming the absence of endothelial (CD31, **b**), pericyte (PDGFRβ, **c**), and microglial (IBA1, **d**) markers in hCOs. Scale bars, 50 μm.

(**e**) Quantitative RT-PCR analysis of hCOs showing expression levels of endothelial (*CD31, CD144*), pericyte (*PDGFRβ*), microglial (*IBA1*), astrocyte (*GFAP*), and pluripotency (*OCT4, NANOG*) markers. Data are shown as mean ± SD (n = 3 from three independent batches).

**Supplementary Figure 10. Characterization of vascularized human cerebral organoids derived from iPSCs (iPSC-vhCOs).**

(**a**) Schematic illustration of stable iPSC^GTM^ line generation and validation.

(**b**) Immunostaining of day-43 iPSC-vhCOs for neuronal (TUJ1+/SOX2+), vascular-glial (CD31+/S100β+), vascular junction (CD31+/ZO1+), and vascular-microglial (CD31+/IBA1+) markers. Scale bars, 50 μm and 75 μm.

(**c**) Flow cytometry analysis of CD31, CD140b, CD11b, and CD68 in day-43 iPSC-vhCOs. Data are shown as mean ± SD (n = 3 from three independent batches).

(**d**) Quantitative RT-PCR analysis of cortical layer markers (*TBR1, CTIP2, SATB2, BRN2, CUX1, REELIN*), forebrain marker (*FOXG1*), choroid plexus marker (*TTR*), endothelial (*CD31*), pericyte (*PDGFRβ*), astrocyte (*GFAP*), and microglial (*IBA1*) markers. Data are shown as mean ± SD (n = 3 from three independent batches).

**Supplementary Figure 11. Vascular permeability assessment of hCOs and vhCOs (related to Fig. 3).**

(**a**) Visualization of FITC-dextran perfusion in hCOs and vhCOs. Organoids were incubated with 40 kDa FITC-dextran for 2 hours. Scale bar, 50 μm.

(**b, c**) Quantification of vascular permeability. (**b**) Mean fluorescence intensity of FITC-dextran in hCOs and vhCOs. (**c**) Permeabilized FITC-dextran concentration. Data are shown as mean ± SD (n = 3 for **b**, n = 5 for **c**, from three independent batches).

(**d**) Representative traces and spike amplitude of electrical activity.

(**e**) Network bursting traces in hCOs and vhCOs.

(**f**) Immunostaining for human-specific CD31 (green) and general CD31 (red) in grafts at 30 d.p.i.

**Supplementary Figure 12. Vascularization enhances cell-cell communication networks in cerebral organoids (related to Fig. 4).**

(**a, b**) Marker genes defining major cell types in hCOs (a) and vhCOs (**b**).

(**c**) Cell type composition and proportional distribution in hCOs and vhCOs (**related to Fig. 4**).

(**d**) Gene expression profiles of signaling pathways involved in neurodevelopment, tissue growth, vascular development, and cell migration (left), with representative signaling pathways in vhCOs highlighted (right).

(**e**) Cell-cell communication networks of key neural developmental and extracellular matrix (ECM) signaling pathways across major cell types in hCOs and vhCOs.

**Supplementary Figure 13. Gene expression analysis of ETV2 and microglial markers in vhCOs (related to Fig. 4).**

(**a**) snRNA-seq analysis showing ETV2 gene expression in day-90 vhCOs.

(**b**) snRNA-seq analysis of microglial marker genes *TREM2, P2RY12, TMEM119*, and *IBA1 (AIF1)* in day-90 vhCOs.

(**c**) snRNA-seq analysis revealing that RUNX1, a key regulator of microglial development, is expressed exclusively in mesodermal (perivascular) lineages and microglia.

(**d**) Quantitative RT-PCR validation of *TREM2, P2RY12*, and *TMEM119* expression in day-90 vhCOs.

**Supplementary Figure 14. Vascularization promotes multi-region neural identity and blood-brain barrier maturation in cerebral organoids (related to Fig. 5).**

(**a**) Mapping of hCO and vhCO datasets onto a human fetal brain reference atlas, revealing enhanced multi-region neural features in vhCOs, particularly in cerebellum, midbrain, and brainstem.

(**b**) Definition of brain region-specific gene sets based on the human fetal brain reference atlas (Braun et al., 2023) (**see Methods**). Two methods were applied: a lenient binarized approach (one region vs. all others) and a stringent paired approach (intersection of all pairwise region comparisons). The binarized method is shown here.

(**c**) GSVA enrichment scores for brain region-specific gene sets (defined by the binarized method) in the neural lineages of hCOs and vhCOs.

(**d**) Mapping of hCO and vhCO datasets onto a human fetal PCW5 gastrula atlas, showing presence scores. hCOs exhibit stronger representation in early fetal tissues.

(**e**) Marker genes defining endothelial subclusters (**related to Fig. 5e**).

(**f**) Marker genes defining astrocyte subclusters (**related to Fig. 5c**).

(**g**) Marker genes defining perivascular subclusters (**related to Fig. 5f**).

**Supplementary Figure 15. Subcluster-level cell-cell communication networks in vhCOs (related to Fig. 5 and 6).**

(**a**) Comparison of outgoing and incoming signaling strengths among major cell types in hCOs, vhCOs, and ZIKV-infected vhCOs (vhCO+ZIKV).

(**b**) Global subcluster-level cell-cell communication network in vhCOs.

(**c**) Outgoing and incoming signaling patterns across subclusters in vhCOs.

(**d**) Contribution of each subcluster to outgoing and incoming signaling within distinct signaling pathways in vhCOs.

(**e**) Identification of signaling patterns in vhCOs using Non-negative Matrix Factorization (NMF).

**Supplementary Figure 16. Blood-brain barrier characteristics in vhCOs (related to Fig. 5).**

(**a**) Contribution of each vhCO subcluster to key vascular barrier-related signaling pathways.

(**b**) Cell-cell communication network within the endothelial subcluster of vhCOs, revealing that tip cells and arterial-type endothelial cells are the primary drivers of vascular migration and angiogenesis.

(**c**) Differential gene expression and GO enrichment analysis comparing AS-Perivascular (defined within the astrocyte subcluster) and Perivascular-AS (defined within the perivascular subcluster) populations, highlighting their distinct molecular signatures and spatial proximity to the vascular barrier.

(**d**) Communication signals from AS-Perivascular and Perivascular-AS populations to the endothelial subcluster. Perivascular-AS cells exhibit greater involvement in extracellular matrix secretion, whereas AS-Perivascular cells are more closely associated with neural-vascular interactions.

**Supplementary Figure 17. Microglia subtype-specific cell-cell communication networks in vhCOs (related to Fig. 5).**

(**a, b**) Activated microglia-specific signaling pathways. (**a**) SPP1 signaling. (**b**) ICAM signaling.

(**c-e**) Migrating microglia-specific signaling pathways. (**c**) PERIOSTIN signaling. (**d**) ANGPTL signaling. (**e**) PDGF signaling. For each pathway: (Left) Chord diagrams illustrating intercellular communication; (Middle) Signal intensity contribution of each ligand-receptor pair; (Right) Functional roles of each participating cell type (bottom) and corresponding gene expression (top).

**Supplementary Figure 18. Inhibition of E-selectin/ICAM-1 impairs barrier function and alters microglial morphology in vhCOs (related to Fig. 3).**

(**a**) TEER measurements of day-90 hCOs, vhCOs, and vhCOs treated with A205804, an E-selectin/ICAM-1 inhibitor. Data are shown as mean ± SD (n = 6 from three independent batches).

(**b, c**) Vascular permeability assay following A205804 treatment. vhCOs were incubated with 40 kDa FITC-dextran for 2 hours, and mean fluorescence intensity was quantified. Scale bars, 50 μm. Data are shown as mean ± SD (n = 3 from three independent batches).

(**d, e**) Immunostaining for IBA1 and CD68 in day-90 vhCOs treated with A205804. (**e**, left) Higher magnification images showing ramified (white arrow) and amoeboid (cyan arrow) microglial morphologies. Scale bars, 50 μm.

**Supplementary Figure 19. Zika virus infection disrupts vascular and neural integrity in vhCOs (related to Fig. 6).**

(**a**) Quantification of organoid size following ZIKV infection of day-90 vhCOs. Data are shown as mean ± SD (n = 3 from three independent batches).

(**b**) Quantification of vhCO (a) and hCO (d) size at 7 days post infection with mock or Zika virus (ZIKV). Data are shown as mean ± SD (n = 3 from three independent batches).

(**c-e**) Immunostaining and quantification of ZIKV-infected day-90 hCOs organoids at 7 days post infection. Co-staining for CD31+/SOX2+, CD31+/NS2B+, TUJ1+/NS2B+, and CD31+/IBA1+ cells. Data are shown as mean ± SD (n = 3 from three independent batches).

**Supplementary Figure 20. Zika virus infection disrupts neural development and neurovascular communication in cerebral organoids (related to Fig. 6).**

(**a**) Mapping of hCO, vhCO, and ZIKV-infected vhCO (vhCO+ZIKV) datasets onto a human neocortex reference atlas. vhCOs exhibit enriched neural characteristics, which are markedly diminished upon ZIKV infection.

(**b**) Distribution of ZIKV genomic reads in single-nucleus sequencing data of vhCO+ZIKV. Abundant free viral genomes suggest extensive cell death.

(**c**) Vascular cells in ZIKV-infected organoids retain fetal brain characteristics rather than adopting adult or tumor-associated phenotypes.

(**d**) Spearman similarity analysis between cell clusters in hCOs, vhCOs, and vhCO+ZIKV. Similarity between High-meta neural progenitor cells (NPCs) and the neural lineage is significantly reduced in vhCO+ZIKV.

(**e**) GSVA enrichment scores for viral infection-associated gene sets in the High-meta NPC subcluster of vhCO+ZIKV, consistent with the normal-to-mesenchymal-to-infection trajectory observed in UMAP (**Relative to Fig. 6k**).

(**f, g**) Differential gene expression (binarized method) and GO enrichment analysis for neurons (**f**) and High-meta NPCs (**g**) across hCOs, vhCOs, and vhCO+ZIKV.

(**h**) Contribution of vhCO+ZIKV subclusters to extracellular matrix (COLLAGEN, LAMININ) and cell migration/immune (CXCL, CD99) signaling pathways.

(**i–l**) Disruption of key neurovascular interaction pathways in vhCO+ZIKV: EPHA (**i**), EPHB (**j**), and CDH2 (**l**).

**Supplementary Figure 21.** Outgoing and incoming signaling communication patterns in vhCO+Z.V.

**Supplementary Figure 22**. Western blot of ETV2 expression after NAEK administration in H9 cells containing GTMs. (n = 3, from three biological replicates) (**Related to Figure 1c**).

**Supplementary Figure 23.** (**a**) Gating strategy (**Related to Figure 2**). (**b**) Flow cytometry analysis of CD31, CD140b, CD11b, and CD68 level at day-30 brain organoid (**Related to Figure 2h**).

**Table S1. Antibodies used in this study.**

| Antibody | Supplier | Catalog. No | Species | Dilution |
| --- | --- | --- | --- | --- |
| IBA1 | Proteintech | 10904-1-AP | Rabbit | 1:200 (IF) |
| SOX2 | CST | 3579S | Rabbit | 1:200 (IF) |
| Synapsin | CST | 5297 | Rabbit | 1:200 (IF) |
| TUJ1 | BioLegend | 801201 | Mouse | 1:200 (IF) |
| PDGFRβ | Proteintech | 13449-1-AP | Rabbit | 1:200 (IF) |
| ZO1 | Proteintech | 21773-1-AP | Rabbit | 1:200 (IF) |
| S100β | Proteintech | 15146-1-AP | Rabbit | 1:200 (IF) |
| GFAP | Abcam | ab302644 | Goat | 1:200 (IF) |
| TMEM119 | Proteintech | 27585-1-AP | Rabbit | 1:100 (IF) |
| P2RY12 | Proteintech | 11976-1-AP | Rabbit | 1:100 (IF) |
| TREM2 | Proteintech | 68723-1-Ig | Rabbit | 1:100 (IF) |
| AQP4 | Proteintech | 16473-1-AP | Rabbit | 1:100 (IF) |
| hNuc | Abcam | ab191181 | Mouse | 1:200 (IF) |
| TBR1 | Abcam | Ab31940 | Rabbit | 1:200 (IF) |
| Brachyury | R&D System | AF2085 | Goat | 1:100 (IF) |
| SSEA4 | R&D System | 962648 | Mouse | 1:200 (IF) |
| ALP | R&D System | 962647 | Mouse | 1:200 (IF) |
| HNF-3β | Santa Cruz | sc-101060 | Mouse | 1:200 (IF) |
| OCT3/4 | R&D System | 962649 | Goat | 1:200 (IF) |
| NANOG | R&D System | 963488 | Goat | 1:200 (IF) |
| NESTIN | BioLegend | 809801 | Mouse | 1:500 (IF) |
| NS2B | GeneTex | GTX133308 | Rabbit | 1:100 (IF) |
| CD31 | Proteintech | 11265-1-AP | Rabbit | 1:100 (IF) |
| CD31 | Abcam | ab9498 | Mouse | 1:200 (IF) |
| CD31-FITC | BD Pharmingen | 555445 | Human | 1:20 (FC) |
| CD140b-PE | BD Pharmingen | 558821 | Human | 1:20 (FC) |
| CD11b-PE | BioLegend | 301306 | Human | 1:20 (FC) |
| CD68-FITC | BioLegend | 333805 | Human | 1:20 (FC) |
| IgG1-PE, κ Isotype | BioLegend | 400114 | Human | 1:20 (FC) |
| IgG1-FITC, κ Isotype | BD Pharmingen | 555748 | Human | 1:20 (FC) |
| IgG2a-PE, κ Isotype | BD Pharmingen | 559319 | Human | 1:20 (FC) |
| IgG2b-FITC, κ Isotype | BD Pharmingen | 555057 | Human | 1:20 (FC) |
| Secondary antibody | Jackson ImmunoResearch | 715-545-151 | Donkey | 1:500 (IF) |
| Secondary antibody | Jackson ImmunoResearch | 711-585-152 | Donkey | 1:500 (IF) |
| Secondary antibody | Jackson ImmunoResearch | 711-165-152 | Donkey | 1:500 (IF) |
| Secondary antibody | Jackson ImmunoResearch | 705-605-147 | Donkey | 1:500 (IF) |

**Table S2. Real time qPCR primers used in this study.**

| **Primer Name** | **Forward 5’-3’** | **Reverse 5’-3’** |
| --- | --- | --- |
| CD31 | GCAGCATCGTGGTCAACATAA | GCAGGACAGGTTCAGTCTTTCA |
| CD144 | AAGGACATAACACCACGAAACG | CAAACTGCCCATACTTGACTGTG |
| OCT4 | CCCCAGGGCCCCATTTTGGTACC | ACCTCAGTTTGAATGCATGGGAGAGC |
| NANOG | TACCTCAGCCTCCAGCAGAT | CCTTCTGCGTCACACCATT |
| TBR1 | GCAGCAGCTACCCACATTCA | AGGTTGTCAGTGGTCGAGATA |
| CTIP2 | GGTGCCTGCTATGACAAGG | GGCTCGGACACTTTCCTGAG |
| SATB2 | CATGCCACAGTCCGCAATG | GGCCCAGAACACAATAGTCTGA |
| BRN2 | CGGCGGATCAAACTGGGATTT | TTGCGCTGCGATCTTGTCTAT |
| CUX1 | GAAGAACCAAGCCGAAACCAT | AGGCTCTGAACCTTATGCTCA |
| REELN | ACATCTACAAGTGTTCAGGCATC | TGGTTACCAAACTGGTGGTCA |
| FOXG1 | CCGCACCCGTCAATGACTT | CCGTCGTAAAACTTGGCAAAG |
| TTR | TGGGAGCCATTTGCCTCTG | AGCCGTGGTGGAATAGGAGTA |
| MME | TGGATCTTGTAAGCAGCCTCA | GCACAACGTCTCCAAGTTGC |
| GLUT1 | GGCCAAGAGTGTGCTAAAGAA | ACAGCGTTGATGCCAGACAG |
| PDGFRb | AGACACGGGAGAATACTTTTGC | AGTTCCTCGGCATCATTAGGG |
| GFAP | AGGTCCATGTGGAGCTTGAC | GCCATTGCCTCATACTGCGT |
| S100b | TGGCCCTCATCGACGTTTTC | ATGTTCAAAGAACTCGTGGCA |
| IBA1 | ATGAGCCAAACCAGGGATTTAC | GGGATCGTCTAGGAATTGCTTGT |
| TMEM119 | CTTCCTGGATGGGATAGTGGAC | GCACAGACGATGAACATCAGC |
| P2RY12 | TTTGTGTGTCAAGTTACCTCCG | CTGGTGGTCTTCTGGTAGCG |
| TREM2 | TCAGGAAGGTCCTGGTGGA | GGGTGGGAAGGGGATTTCTC |
| ETV2 | ACTAACCACCGAGGTCCCAT | TTCATGCCCGGCTTTCTCTT |
| MmPylRS | GGCAAACGAAGACCAGACAA | CTGTATTCTCAAGAGGTTTC |
| tRNA^pyl^ | GACTCTAAATCCGTTCAGCCGGG | AAAATGGCGGAAACCCCGG |
| 18S RNA | GGTGCATGGCCGTTCTTA | TCGTTCGTTATCGGAATTAACC |
| GAPDH | GGAGCGAGATCCCTCCAAAAT | GGCTGTTGTCATACTTCTCATGG |
